# DNA Methylation-Driven Genes for Developing Survival Nomogram for Low-Grade Glioma

**DOI:** 10.3389/fonc.2021.629521

**Published:** 2022-01-17

**Authors:** Yingyun Guo, Yuan Li, Jiao Li, Weiping Tao, Weiguo Dong

**Affiliations:** ^1^ Department of Gastroenterology, Renmin Hospital of Wuhan University, Wuhan, China; ^2^ Department of Oncology, Renmin Hospital of Wuhan University, Wuhan, China

**Keywords:** low-grade glioma, DNA methylation-driven genes, TCGA database, CGGA database, survival nomogram model, prognosis

## Abstract

Low-grade gliomas (LGG) are heterogeneous, and the current predictive models for LGG are either unsatisfactory or not user-friendly. The objective of this study was to establish a nomogram based on methylation-driven genes, combined with clinicopathological parameters for predicting prognosis in LGG. Differential expression, methylation correlation, and survival analysis were performed in 516 LGG patients using RNA and methylation sequencing data, with accompanying clinicopathological parameters from The Cancer Genome Atlas. LASSO regression was further applied to select optimal prognosis-related genes. The final prognostic nomogram was implemented together with prognostic clinicopathological parameters. The predictive efficiency of the nomogram was internally validated in training and testing groups, and externally validated in the Chinese Glioma Genome Atlas database. Three DNA methylation-driven genes*, ARL9, CMYA5*, and *STEAP3*, were identified as independent prognostic factors. Together with *IDH1* mutation status, age, and sex, the final prognostic nomogram achieved the highest AUC value of 0.930, and demonstrated stable consistency in both internal and external validations. The prognostic nomogram could predict personal survival probabilities for patients with LGG, and serve as a user-friendly tool for prognostic evaluation, optimizing therapeutic regimes, and managing LGG patients.

## Introduction

Glioma is a central nervous system tumor derived from glial cells and is the most prevalent primary malignant intracranial tumor (1, 2). According to statistics from the Central Brain Tumor Registry of the United States (CBTRUS), gliomas account for approximately 27% of all central nervous system tumors, and 80% of brain malignancies, with 15,000-17,000 new cases in the United States per year (3). Gliomas are traditionally divided into lower-grade gliomas (LGG) and glioblastoma multiforme (GBM). GBM is one of the most frequently diagnosed malignant gliomas, and its characteristics include highly aggressive progression and short median overall survival (OS) of 12-16 months (4, 5). Although LGG is less invasive than GBM, it nevertheless causes considerable morbidity, and presents a difficult challenge to doctors due to heterogeneity in their clinical behavior (2, 6, 7). Current predictive models for LGG are either unsatisfactory or not user-friendly, which greatly hinders clinical application. Therefore, a reliable and user-friendly predictive model for LGG patient prognosis is urgently needed.

Epigenetic alterations have been reported to play crucial roles in cancer development, and aberrant DNA methylation is one of the most well-characterized epigenetic modifications, and is of paramount importance (8). DNA methylation plays a key role in transcriptional regulation and maintains genome stability without changing the DNA sequence (9). In particular, a large number of studies have demonstrated that DNA methylation alterations can make available significant information for early tumor diagnosis and prognostic prediction (10–12).

Moreover, there is a growing number of studies focusing on abnormal DNA methylation, which is viewed as a key factor in the occurrence and progression of glioma (13–16). For example, Chen et al. (17) showed that an MGMT methylation group exhibited prolonged progression-free survival (PFS) compared to the negative MGMT methylation group. Wang et al. (18) reported that eight genes affected by DNA methylation modification have independent prognostic values for GBM patients. Therefore, identifying novel genes with aberrant DNA methylation in LGG is critical to gain better insights into the biological mechanisms involved in LGG, thereby offering a promising tool for effective prognostic prediction.

A nomogram is a graphical representation of logistic regression or Cox regression, which can be employed to predict the survival or diagnosis probability of individuals with high accuracy and good clinical practicability (19, 20). Although an association between DNA methylation alterations and prognosis in LGG patients has been reported, most studies have been based either on gene expression or methylation, and no study has combined gene expression/methylation with significant clinical features to establish a survival nomogram for LGG patients (21). In this study, we sought to set-up and independently validate a nomogram incorporating multiple parameters for survival estimation among patients with LGG. The findings of our study will help further improve individualized management for patients with LGG.

## Methods

### Data Curation From TCGA and CGGA Databases

RNA sequencing and DNA methylation sequencing data in LGG and accompanying clinicopathological parameters were downloaded from The Cancer Genome Atlas (TCGA) database (22) (https://portal.gdc.cancer.gov/). Samples with incomplete data were removed, and finally, 516 LGG patients were included in this study. To verify the prognostic value of selected genes and the final nomogram, 104 LGG patients with expression and methylation array data, and 620 LGG patients with RNA sequencing data, were curated from the Chinese Glioma Genome Atlas (CGGA) database (23)(www://cgga.org.cn/). Data were utilized according to the data access policy of TCGA and CGGA. All analyses were conducted in accordance with relevant regulations and guidelines.

The following clinical information was collected from the TCGA databases: patients’ age, sex, tumor grade (WHO grade I or WHO grade II), histological type (astrocytoma, oligoastrocytoma and oligodendroglioma), date of initial pathologic diagnosis (1993–2013), age at diagnosis (14–86), race (white, black or African American, Asian, American Indian or Alaskan native), OS, and survival status (alive or dead), were also retrieved, where available. 

### Differential Expression Analysis in LGG

Among the 516 LGG patients represented in TCGA data, 14 had both primary and recurrent samples. Differential expression analysis was performed among the 502 primary tissues of LGG patients without recurrence, and 14 samples with recurrence using Student’s *t*-test followed by p value adjustment by the “Benjamini-Hochberg” method, utilizing R software (v.3.6.1). Differentially expressed genes (DEGs) were defined as being significantly up or downregulated when p values were < 0.001 and absolute log2 fold-change (LFC) > 1.

### DNA Methylation Correlation Analysis

Here, single specific gene DNA methylation values were estimated from mean DNA methylation Beta values for complete CpG sites in gene promoters. In order to recognize gene transcription and predict DNA methylation, gene expression and DNA methylation data were robot-like matched. Relationships between gene expression levels and extent of DNA methylation were investigated in 516 LGG samples. Methylation-associated genes (Methygenes) were defined as |Coef| > 0.5 and p value < 0.001.

### Survival Analysis and LASSO Regression

To avoid the influence of interventions on OS, we excluded those patients with OS < 30 days, left with 481 LGG individuals included in the survival analysis. Univariate Cox regression was performed to uncover survival-associated genes (Survgenes) in LGG patients. The best cut-off value for each gene was determined using the survminer package (v.0.4.6), and significant Survgenes were defined as having p values < 0.001.

Candidate genes were selected using Venn diagrams; only genes meeting the criteria of significance in differential expression, OS, and methylation correlation analyses, were chosen for downstream analysis. LASSO (least absolute shrinkage and selection operator) was performed to evaluate potential correlations involving DNA methylation-driven gene expression levels and patient prognoses in order to identify independent DNA methylation-driven genes related to prognosis in the TCGA dataset.

### Establishment and Validation of Predictive Model

To compare and further improve the predictive efficiency of the model, survival analyses were also performed involving clinical features so as to identify significant prognostic factors. A risk score prediction model was constructed based on the expression levels of three DNA methylation-driven genes filtered by LASSO, together with age, sex, and *IDH1* mutation data.

For internal validation, patients were randomly divided into a training set (n = 289) and a test set (n = 192) to validate the predictive capability of the prognostic model. In the whole set, time-dependent receiver operating characteristic (tROC) curves using the survivalROC package (v.1.0.3) (24) were employed to compare the predictive efficiency of the individual factors and model.

### Building and Validating Prognostic Nomogram

To present a predictive model with integrated factors in a user-friendly way, a nomogram was built using the rms R package (v.5.1-4) (25). Validation using calibration curves was then performed. Calibration of the nomogram was evaluated using calibration curves, graphically assessed with the relationships between the actual observed rates and the probabilities predicted by the nomogram, by which the 45° line indicates the best prediction. To calculate the discrimination accuracy of our nomogram, concordance index (C-index) was measured. The radiomic nomogram was submitted to bootstrapping validation (1,000 bootstrap resamples) to compute a relatively corrected C-index.

### External Validation Analysis

Because different detection platforms were used in TCGA and CGGA databases, methylation levels of *CMYA5* and *ARL9* were not available in the CGGA database. Therefore, we attempted to verify the power of each factor and the model with available data in CGGA. The cut-off values of the high and low groups for each gene were determined by quantiles set as in TCGA dataset. KMplots (Kaplan-Meier Plotter) for each gene and models with three genes, with or without clinical parameters, were provided.

### Statistical Analysis

All statistical analyses were performed using R software (v.3.6.1). The Cox regression model was applied to evaluate the significance of each clinical parameter on OS. Survival curves were generated using Kaplan–Meier plots, and compared using the log-rank test. Time-dependent receiver operating characteristic (tROC) curves generated using the survivalROC package (v.1.0.3) (24) were employed to measure predictive power; the area under the curve at different time points (AUCt) could be determined and compared easily. All statistical analyses were 2-sided, and probability values of p < 0.05 were considered statistically significant.

## Results

### Identification of Methylation-Driven Genes in LGG

**Figure 1:**
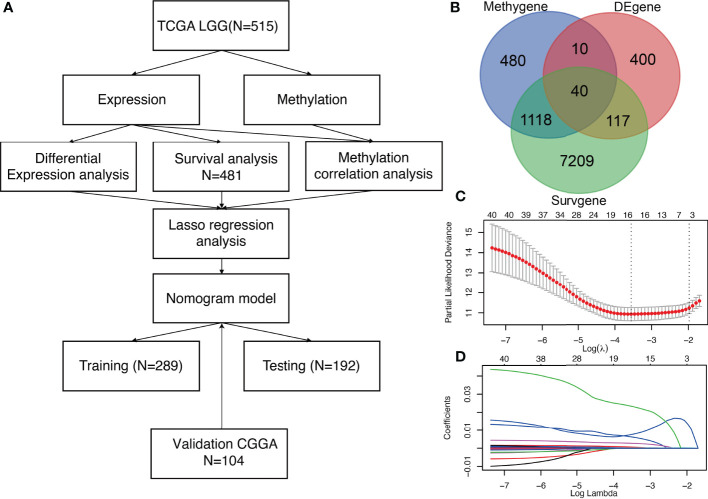
Identification of three significant prognostic genes in LGG and LASSO results **(A)** Technical roadmap for the whole study. **(B)** Venn diagram of 40 OCGs. **(C)** Partial likelihood deviance for LASSO regression. **(D)** LASSO regression analysis of 40 genes in LGG.

An analysis pipeline of this exploration is shown in **Figure 1A**. First, DEG analysis was performed involving primary tissues of LGG patients without recurrence (n = 502), and those with recurrence (n = 14) in TCGA (**Figure 1A**). Using a cut-off criterion of p < 0.001 and |log2 FC| > 1, 567 genes were identified as DEGs (**Figure 1B**), which were differentially expressed between patients with recurrence and patients without recurrence. Secondly, methylation correlation analysis revealed a total of 1,685 Methygenes whose expression was significantly different with changes in DNA methylation levels. (**Figure 1B**). Thirdly, 8,484 Survgenes (p < 0.001) were identified by survival analysis in the 481 LGG samples (**Figure 1B**). Furthermore, 40 overlapping candidate genes (OCGs) were identified using Venn diagrams (**Figure 1B**) involving genes identified in the above steps.

**Figure 2:**
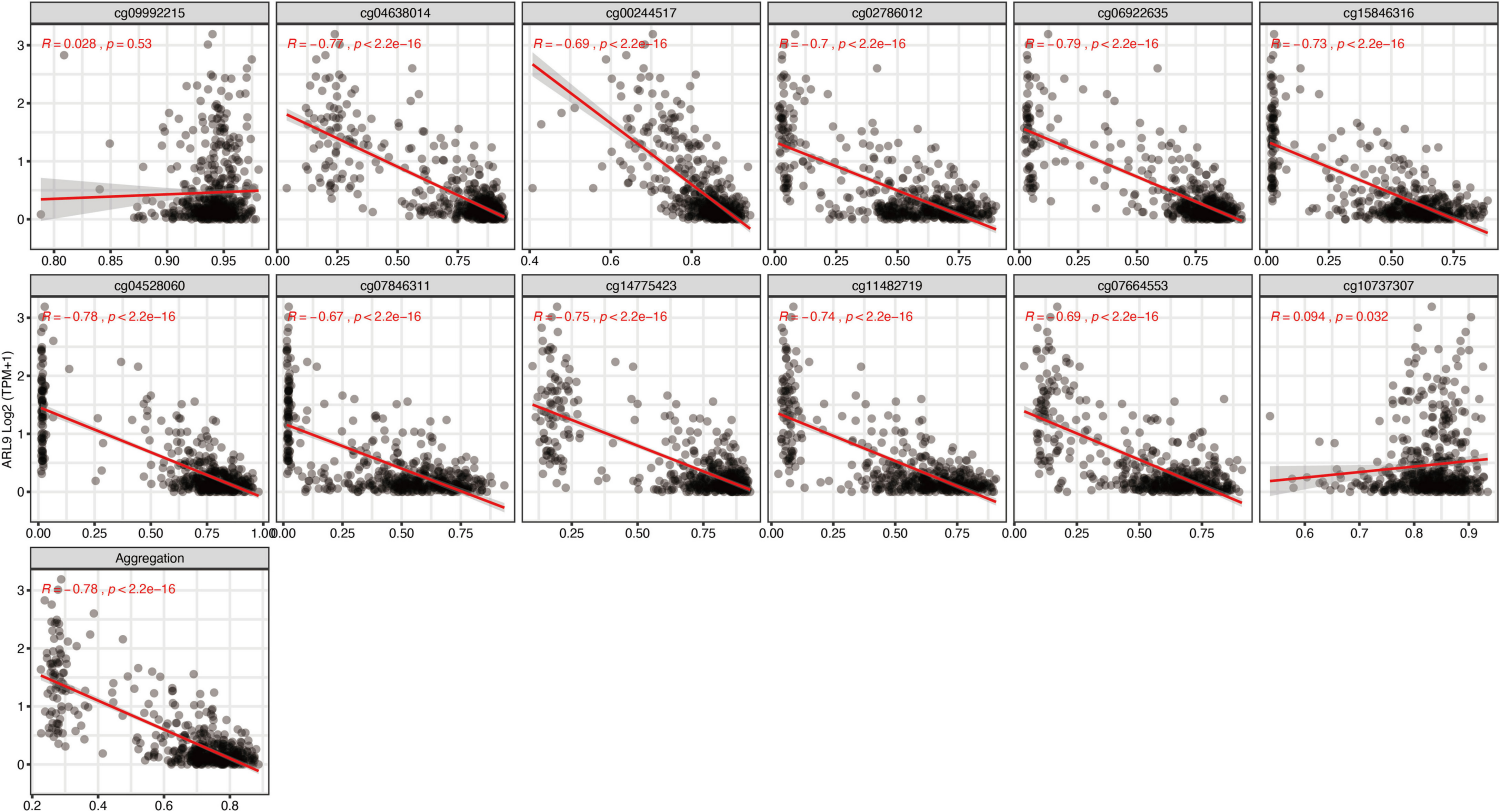
Regression analysis involving each specific CpG site and aggregated levels, and the expression of *ARL9* in the whole dataset from TCGA.

**Figure 3:**
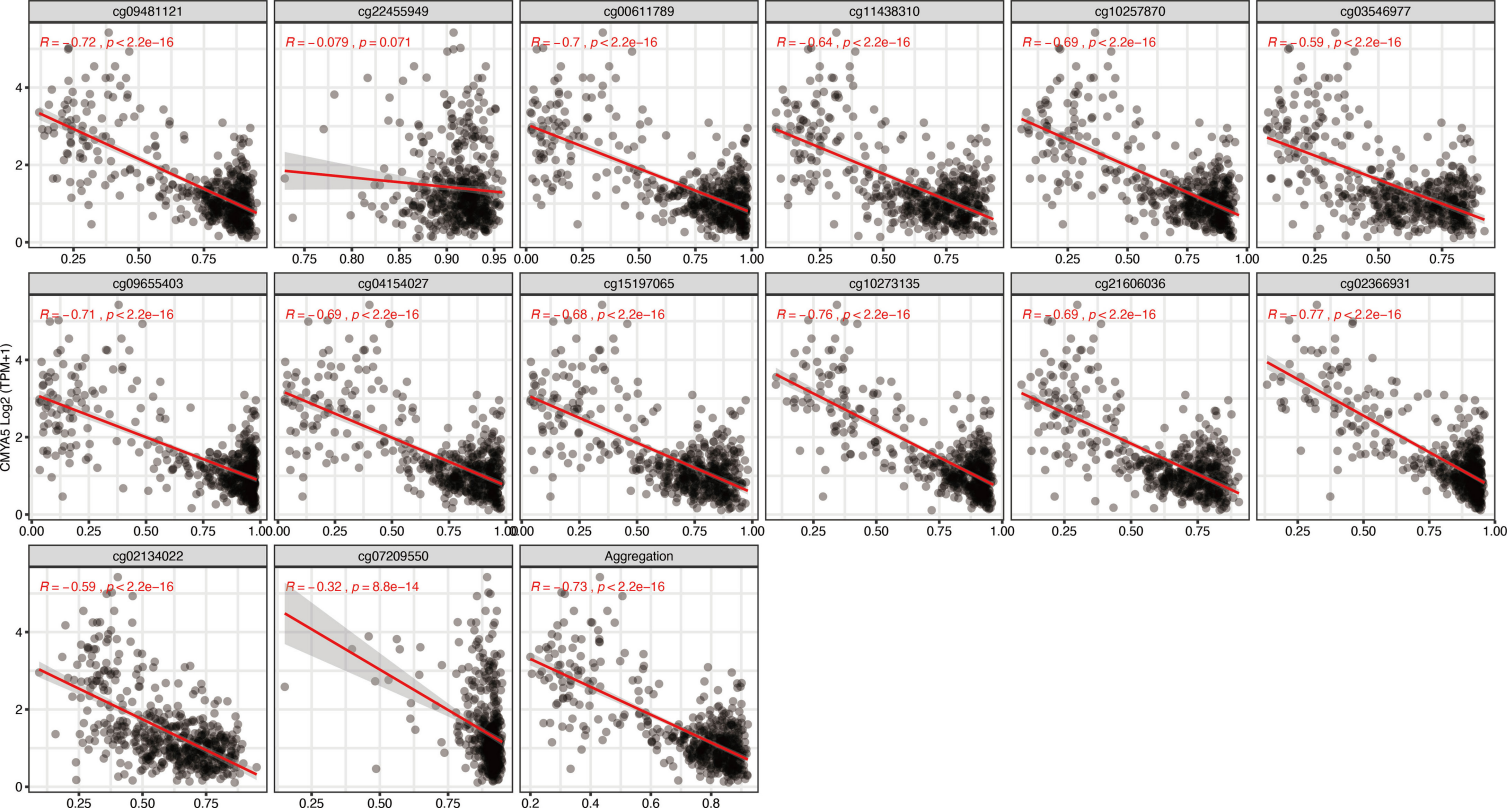
Regression analysis involving each specific CpG site and aggregated levels, and the expression of *CMYA5* in the whole dataset from TCGA.

**Figure 4:**
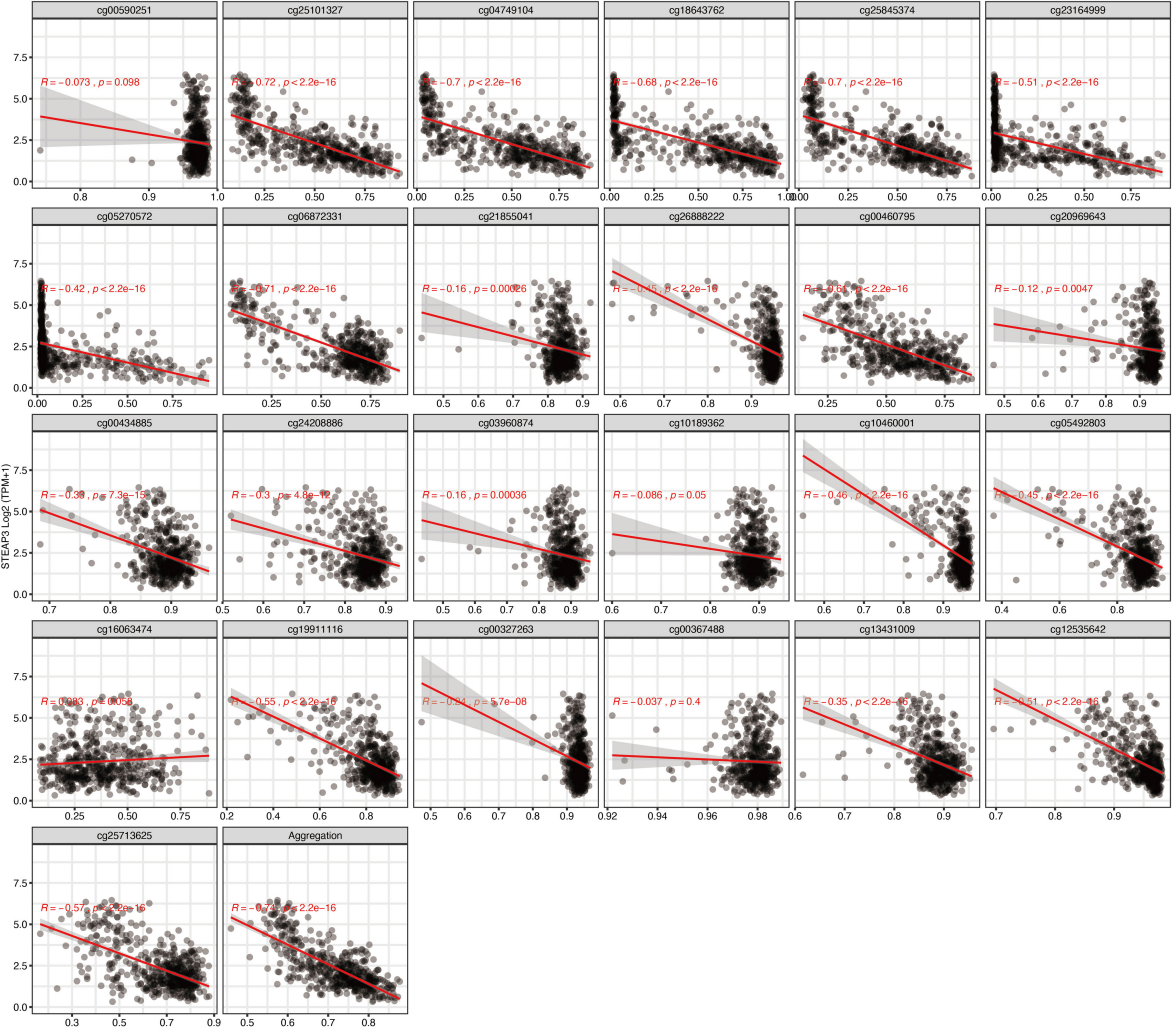
Regression analysis involving each specific CpG site and aggregated levels, and the expression of *STEAP3* in the whole dataset from TCGA.

To further narrow down and uncover the driving factors involving the 40 OCGs, LASSO analysis was performed and *ARL9, CMYA5*, and *STEAP3* were identified as driving factors related to OS (**Figures 1C, D**). Correlations between each specific CpG site and expression of the three genes are shown. Generally, the expression of each gene was negatively correlated with the methylation levels of almost all CpG sites, and the aggregated level (**Figures 2**–**4**).

### Establishment of Nomogram Prognostic Model

**Figure 5:**
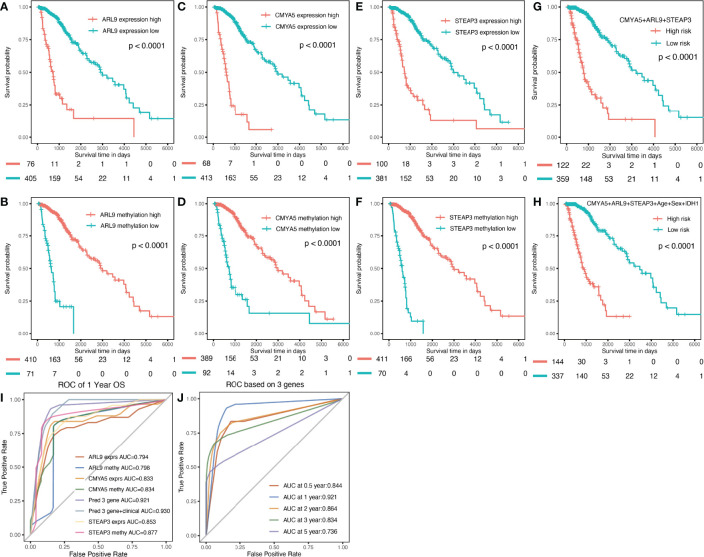
Prognostic analysis of three genes individually and in combination for the whole TCGA dataset. **(A, C, E)** K-M OS curves based on expression levels for the three genes. **(B, D, F)** based on DNA methylation data. **(G)** based on expression and methylation data. **(H)** based on 3-gene signature with clinical parameters. **(I)** Multi-index ROC curve of indicators **(J)** Time-dependent ROC analysis the of the 3-gene model.

Survival analyses were performed based on the gene expression and DNA methylation levels of *ARL9, CMYA5*, and *STEAP3* in the cohort of LGG patients from TCGA data. High levels of *ARL9* (**Figures 5A, B**), *CMYA5* (**Figures 5C, D**), and *STEAP3* (**Figures 5E, F**) expressions, and DNA hypomethylation of the three genes were significantly associated with poorer prognosis, indicating that DNA methylation is involved in the regulation of gene expression, and that the control relationship may be negative (**Figures 2**, **3**). Meanwhile, age, sex, and *IDH1* mutation status were also significant in the OS analysis. Multivariate Cox proportional hazards regression analysis was performed to establish prognostic models with or without clinical factors. KMplots of the two prognostic models are shown in **Figures 5G, H**. Intuitively, adding clinical parameters, including age, sex, and *IDH1* mutation status, did not dramatically improve the predictive efficiency of the prognostic model (**Figures 5G, H**). Furthermore, we conducted ROC curve analyses for specificity, sensitivity, and predictive value of the prognostic parameters assessed. At 1-year OS, the time-dependent AUC of the 3-gene model was 0.921 (**Figure 5I**), indicating high performance in predicting OS in LGG patients. The AUC of the model with three genes and clinical parameters was 0.930, which was slightly higher than that of the 3-gene model alone (**Figure 5I**), in which each was higher than the individual factors (**Figure 5I**). The AUCs of time-dependent ROC analysis at 0.5-, 1-, 2-,3-, and 5-year OS of the 3-gene model in the whole set were 0.844, 0.921, 0.864, 0.834, and 0.736, respectively (**Figure 5J**). In conclusion, the 3-gene model performed well in predicting OS of LGG patients.

**Figure 6:**
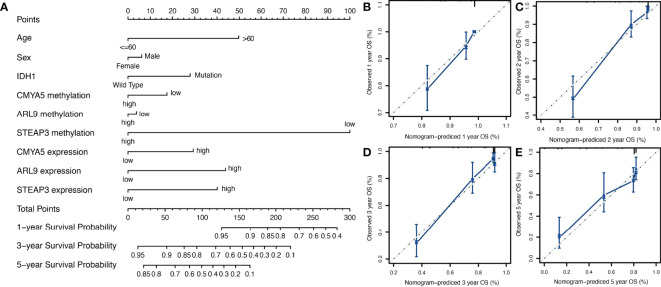
Nomogram construction and validation. **(A)** Prognostic nomogram to predict 1-, 3-, and 5-year survival probability for patients with LGG. **(B–E)** Calibration curves of the nomogram for predicting the probability of OS at 1-, 2-, 3-, and 5-years. The x-axis represents nomogram-predicted probabilities; the y-axis represents actual survival rates of patients.

To provide a more user-friendly clinical predictive model, a prognostic nomogram was built with the three DNA methylation-driven genes and clinicopathological factors. Using the survival nomogram, the proportion of patients with probabilities of 1-, 3-, and 5-year survival times can be reliably predicted (**Figure 6A**). *STEAP3* methylation was the dominant factor in the nomogram (**Figure 6A**). Moreover, calibration curves for survival prediction demonstrated that the nomogram predictive outcome showed good agreement with actual observations in 1-, 2-, 3-, and 5-year OS rates (**Figures 6B**–**E**). In summary, the final prognostic nomogram exhibited high prediction efficiency and good consistency in LGG patients.

### Internal Validation of the Prognostic Nomogram

**Figure 7:**
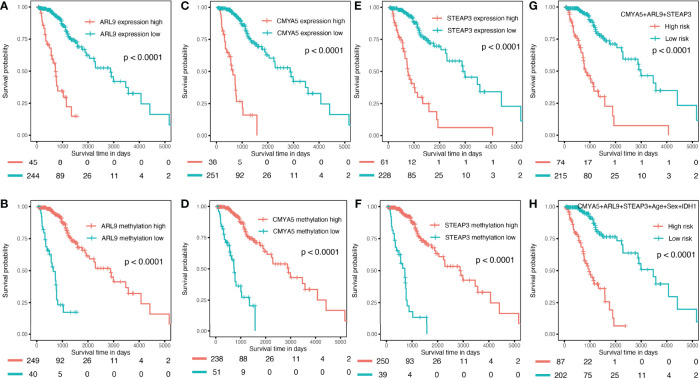
Validation in the internal training set. **(A–F)** K–M OS curves for 3-gene expression and methylation in the internal training set. **(G)** combination of 3 genes. **(H)** of the nomogram.

**Figure 8:**
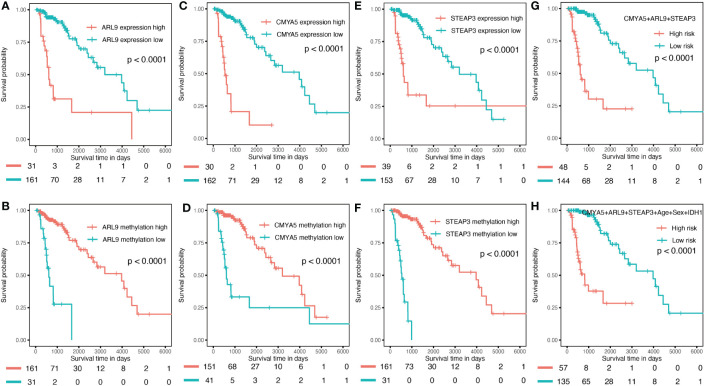
Validation in the internal validation set. **(A–F)** K–M OS curves for 3-gene expression and methylation in the internal validation set. **(G)** combination of three genes. **(H)** of the nomogram.

To verify the predictive capability of the prognostic nomogram, 481 LGG patients were randomly divided into a training set (n = 289) and a testing set (n = 192) by 6:4 ratio. Patients were categorized into two groups: low-risk and high-risk, with the same cut-off value as in the previous analysis. Specifically, KMplots for each gene demonstrated similar patterns in the training (**Figures 7A**–**F**) and testing sets (**Figures 8A**–**F**), which were largely consistent in the whole cohort. Moreover, survival analysis revealed that patients with high scores in the risk model had significantly shorter OS than those in the low score group (**Figures 7G, H**, **8G, H**), suggesting that the prognostic model of the nomogram was influenced by randomization, and showed high consistency in TCGA LGG cohort.

### External Validation of the Prognostic Nomogram

**Figure 9:**
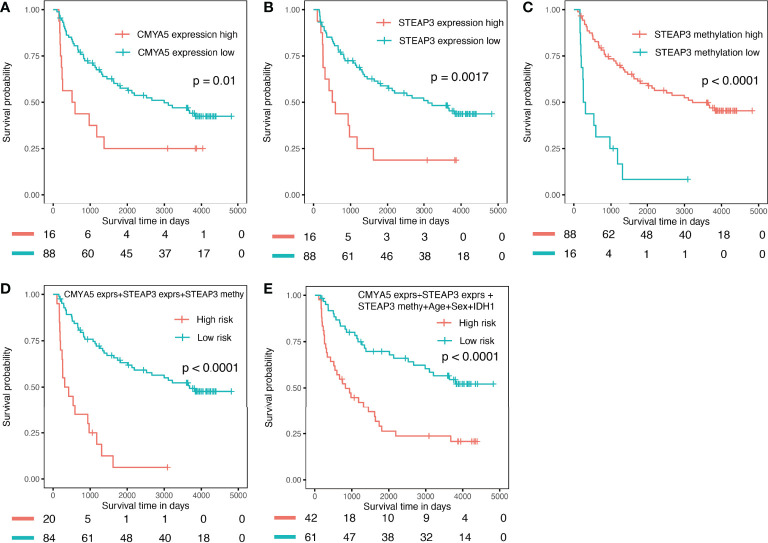
Validation in the external CGGA validation dataset. **(A)** K–M OS curves of *CMYA5* expression **(B, C)** of *STEAP3* expression and methylation. **(D)** combination of *CMYA5* expression, *STEAP3* expression and methylation. **(E)** combination of *CMYA5* expression, *STEAP3* expression and methylation with age, sex, and *IDH1* status.

The CGGA dataset was used for external validation of the prognostic nomogram; however, methylation levels of *CMYA5* and *ARL9* and expression data for *ARL9* were not available because different detection platforms were used in TCGA and CGGA databases. Therefore, we attempted to verify the power of each factor and the model with available data in CGGA. As shown in **Figure 9**, high expression of *CMYA5* and *STEAP3* predicted poorer OS, while high methylation of *STEAP3* indicated better OS (**Figures 9A**–**C**). A multivariate prognostic model employing the available factors also demonstrated high predictive efficiency (**Figure 9D**), while adding clinical parameters enriched more patients with high risk (**Figure 9E**). In summary, these results demonstrated that it was reliable to create prognostic models based on these three DNA methylation-driven genes.

## Discussion

In the present study, we first constructed a prognostic prediction nomogram based on three DNA methylation-driven genes and clinical parameters. Predictive efficiencies were evaluated and compared in two cohorts, including 516 glioma patients from TCGA database, and 104 patients from the CGGA database. The AUC of the final prognostic nomogram was 0.930, which was higher than for each factor individually. These results indicated that the prognostic nomogram was powerful in predicting OS of LGG patients.

To date, several prognostic models for glioma have been reported. Wang et al. (26) constructed a risk score model using five selected pseudogenes (*ANXA2P2, EEF1A1P9, FER1L4, HILS1*, and *RAET1K*) in glioma patients, and Zeng et al. (27) developed and validated a 3-gene (*EMP3, GSX2*, and *EMILIN3*) prognostic signature in LGG cases by combining multi-dimensional genomic data from TCGA and CGGA datasets, the two predictive models based only on gene alterations with no clinical parameters. The AUCs for 1-year survival of Wang studies were 0.862, which is lower than our prognostic nomogram, demonstrated an AUC of 0.930. In addition, the models of Cheng et al. (28) and Gittleman et al. (29) are only suitable for primary glioblastoma, lacking predictive power for other types of glioma; in addition, only a small number of studies are available for WHO I and WHO II glioma models (30, 31). Zhao et al. (32) constructed a prognostic model for GBM survival prediction based on methylation-driven genes. The AUC of the validation set was 0.808, which was also less effective than our prognostic nomogram. Therefore, our nomogram for LGG patients included not only three methylation-driven genes, but also other clinically important variables related to prognosis; it demonstrated higher predictive efficiency than existing models, and was more user-friendly.

Recent studies have demonstrated that there are various survival-associated genes with epigenetic abnormalities in gliomas (33–35). Furthermore, DNA methylation is a frequent type of epigenetic change; it is stable and easily detectable through high-throughput and sensitive equipment requiring minimal glioma samples (36). Therefore, identifying novel DNA methylation-driven genes is urgently needed. In the current study, all the three DNA methylation-driven genes were confirmed to be prognosis-related genes, with negative relationships. Han et al. (37) demonstrated that *STEAP3* is overexpressed in glioma samples and validated to be related to poorer clinical prognosis in glioma patients. As a member of the iron regulatory protein family, *STEAP3* plays a critical role in iron uptake (38). In addition, the function of STEAP family for the prognosis prediction of GBM and other types of human cancer s have been validated in several studies (39, 40). Previous research has established that disorders involving iron metabolism play important roles in tumorigenesis, and iron uptake by glioma stem cells (GSCs) can be increased (38, 41). Certain studies have concluded that it may be caused by *STEAP3* activating the TfR-STAT3 pathway in GBM, and that knockdown of the transferrin receptor (TfR) significantly influences the impact of *STEAP3* overexpression on malignant phenotypes in GSCs (42, 43). These two crucial factors involving iron regulatory-TfR and ferritin are also vital for the proliferation of GSCs, and for tumor growth *in vivo* (43). However, researchers still need to explore the potential clinical practice and role of *STEAP3* in the progression of human gliomas. In contrast to *STEAP3*, the roles of *ARL9* and *CMYA5* in glioma have rarely been reported and remain obscure. Tan et al. (44) shown that *ARL9* is negatively regulated by ARL9 methylation, and both low ARL9 expression and hypermethylation predicted favorable OS and PFS in LGG patients. Its expression exhibited a close correlation with some immune cells, especially CD8+ T cells, indicating that probably plays an important role in immune cell infiltration in LGG. Prior studies have reported that the difference in *CMYA5* expression levels were detected as a potential driven gene in Taiwanese patients with endometrial cancer (45).

Previously, isocitrate dehydrogenase (*IDH*) mutations were first reported in 2008 by Parsons et al. (46) after GBM exome sequencing. Since the 2016 WHO reclassification of gliomas, it is thought that molecular alterations, such as 1/2 (*IDH*) mutations, are significantly more important than the WHO grading score. Song et al. (47, 48) showed that in comparison to patients with *IDH* wild-type glioma, patients with *IDH1*-mutated grade III tumors had better chemotherapy responses and improved prognoses. To date, mutations involving the *IDH1* gene represent the most common alterations in LGG patients, and are significantly related to better prognosis (49, 50). Most studies have demonstrated that *IDH1* mutation plays a key role in the tumorigenesis and progression of glioma by DNA hypermethylation, histone hypermethylation, hypoxia-inducible factor-1a level changes, and oxidative stress mechanisms (51, 52). For the purposes of improving the model’s prognostic prediction power based on the above 3 DNA methylation-driven genes, a more user-friendly and highly accurate predictive nomogram was established by combining traditional clinical prognostic indicators (including *IDH1* status, age, and sex). All of these three indicators were validated to be independent prognostic factors in terms of OS of glioma patients after examination by Cox model analysis.

It is worth noting that there were several limitations to our study. First, the ethnicity of the LGG patients from TCGA and CGGA databases were different, which might have influenced the results. Second, the establishment and validation of our prognostic model was based on public datasets and different detection methods were used; some data were not available in CGGA due to these issues. Third, the cohorts were also relatively small, and need to be validated in larger, multicenter, and prospective clinical cohorts. Notwithstanding these limitations, this study provides a readily-available nomogram for clinical practice, and opens a new door for methylation-driven gene applications, which may be beneficial to LGG patients. Further research should also be conducted to determine the effectiveness of this nomogram, and possible new strategies for targeted therapy.

In summary, our study identified three methylation-driven genes, namely *ARL9, CMYA5*, and *STEAP3* and, combined with clinical factors, we first established and independently validated a prognostic nomogram to provide novel and user-friendly options for prognostic evaluation of LGG patients, and to improve their treatment.

## Data Availability Statement

The datasets presented in this study can be found in online repositories. The names of the repository/repositories and accession number(s) can be found in the article/supplementary material.

## Author Contributions

YL initiated the hypothesis and organized the studies. YL, YYG, and JL analyzed the data and contributed to edit the manuscript. YYG and YL participated in reviewing and modifying the manuscript. All authors have equally involved in study design and drafting of the manuscript. All authors contributed to the article and approved the submitted version.

## Funding

This work was supported by the National Natural Science Foundation of China (Grant 81902369).

## Conflict of Interest

The authors declare that the research was conducted in the absence of any commercial or financial relationships that could be construed as a potential conflict of interest.

## Publisher’s Note

All claims expressed in this article are solely those of the authors and do not necessarily represent those of their affiliated organizations, or those of the publisher, the editors and the reviewers. Any product that may be evaluated in this article, or claim that may be made by its manufacturer, is not guaranteed or endorsed by the publisher.
